# The Survival and Success of Teeth With External Cervical Resorption: A Multi‐Centre, Retrospective Study

**DOI:** 10.1111/iej.70172

**Published:** 2026-06-04

**Authors:** Shanon Patel, Ryan Walsh, Paula Villa, Brent Hiebert, Elizabeth Shin Perry, Rahul Sadhwani, Nassr Al‐Nuaimi

**Affiliations:** ^1^ Faculty of Dentistry, Oral & Craniofacial Sciences King's College London London England UK; ^2^ Guy's & St Thomas NHS Foundation Trust London England UK; ^3^ Specialist Practice London England UK; ^4^ Specialist Practice Dallas Texas USA; ^5^ Texas A&M University College of Dentistry Dallas Texas USA; ^6^ Universidad de Antioquia Medellín Colombia; ^7^ Specialist Practice Medellín Colombia; ^8^ Specialist Practice Oregon USA; ^9^ Harvard School of Dental Medicine Boston Massachusetts USA; ^10^ Private Practice Massachusetts USA; ^11^ Tufts University School of Dental Medicine Boston Massachusetts USA

**Keywords:** external cervical resorption, prognostic factors, success rates, survival rates

## Abstract

**Objectives:**

To evaluate the survival and success outcomes of teeth affected by external cervical resorption (ECR) managed using three distinct treatment strategies, with periapical radiographs and small field of view cone beam computed tomography used for diagnosis and treatment planning.

**Materials and Methods:**

This retrospective, multi‐centre cohort study analysed 296 teeth diagnosed with ECR across five specialist endodontic practices between 2009 and 2024. Management approaches included periodic review (Treatment 1), external repair with or without endodontic intervention (Treatment 2), and internal repair (Treatment 3). Treatment selection was based on clinical and radiographic characteristics, including lesion accessibility, symptomatology, and the presence of bone‐like tissue. Patients underwent annual clinical and radiographic review. The primary outcome was tooth survival; secondary outcomes included success (asymptomatic and healed) and failure (extraction due to progression or non‐restorability). Kaplan–Meier survival estimates and Cox proportional hazards models were applied.

**Results:**

A total of 296 teeth from 233 patients were followed for a mean of 2.9 years (range: 12–123 months). Estimated cumulative survival rates at 1, 2, 5, 7, and 10 years were 95.5%, 93.3%, 85.7%, 80.5%, and 80.5%, respectively. Multivariable analysis demonstrated that internal repair and higher Patel lesion classifications, particularly circumferential involvement greater than 180°, were associated with a higher extraction hazard. The Heithersay classification was not significantly associated with survival. Estimated cumulative survival rates were 93.6%, 90.2%, and 59.1% for Treatments 1, 2, and 3, respectively.

**Conclusions:**

Higher estimated survival was observed among teeth selected for periodic review, followed by external repair and internal repair. Greater three‐dimensional lesion extent, > 180°, was associated with an increased extraction hazard. Treatment selection was influenced by lesion characteristics and clinical judgement; differences between treatment groups should be interpreted as associations rather than evidence of treatment superiority. External repair remained viable, whereas internal repair was associated with a greater risk of failure. The Patel (3D) classification was more strongly associated with survival outcomes than the Heithersay (2D) classification and supports detailed lesion characterization and treatment planning. Treatment decisions should be guided by lesion extent, accessibility, symptoms, and evidence of reparative activity. The findings represent associations observed within a retrospective cohort.

## 1 Introduction

External cervical resorption (ECR) usually occurs in the cervical region of a tooth (Irinakis et al. 2022; Mavridou et al. 2022). It is caused by localized damage and/or disturbance of the periodontal ligament and the underlying non‐mineralized (pre‐) cementum, resulting in the exposed dental hard tissues being resorbed by odontoclasts (Luso and Luder 2012); in advanced cases the root canal system may be affected (Mavridou et al. 2017). ECR may be destructive (resorptive) in nature, and/or in advanced cases may be reparative in nature, with bone‐like tissue replacing the previously resorbed area of the tooth (Patel et al. 2018).

The prevalence of ECR is reported to be between 0.96%–2.3% (Irinakis et al. 2020; Huang et al. 2023). The increasing reported prevalence of ECR may, in part, reflect greater diagnostic awareness and the wider use of CBCT over the last two decades (Huang et al. 2023). The aetiology of ECR is unknown, however, several potential predisposing factors have been reported to be linked to its development. The most associated factors are orthodontic treatment, dental traumatic injuries, parafunctional habits and cat ownership (Mavridou et al. 2017; DeLuca et al. 2023; Patel et al. 2025).

Timely and effective treatment is desirable to prolong the longevity of the tooth as well as to alleviate and/or prevent symptoms. The aims of active treatment are to excavate the resorptive tissue, restore the resorptive defect and monitor the affected tooth for recurrence, however, in some cases periodic reviews, i. e. no active treatment may be preferable (Bardini et al. 2023). The management of ECR is dictated by several factors including symptoms, extent and accessibility of the lesion, as well as the presence of bone‐like reparative tissue (Mavridou et al. 2022). Treatment options include external repair with or without endodontic treatment, internal repair with endodontic treatment, intentional replantation, and periodic reviews (Bardini et al. 2023; Patel et al. 2023).

There is limited data on the prognosis of different management strategies for ECR (Irinakis et al. 2022). Heithersay published the first extensive study to document treatment outcomes of ECR based on 101 teeth with a minimum follow‐up of 3 years. Based on the Heithersay classification, the survival rates were 100% (class 1/2), 96.8% (class 3), and 50% (class 4) survival, respectively (Heithersay 1999). In that study, radiographic examination was limited to periapical radiographs (PR) as at the time it was the only relevant, contemporaneous imaging technique available for management and follow‐up. However, PR have inherent limitations in accurately diagnosing the presence and nature of ECR or may result in misdiagnosis as internal root resorption.

It is well documented that cone beam computed tomography (CBCT) is more accurate for confirming the presence of ECR compared to PR, including assessment of the apico‐coronal and circumferential extent of the ECR, and the presence and extent of bone‐like tissue (Patel et al. 2018; Rodriguez et al. 2017). This crucial information is essential to determine the viability of active treatment of ECR, as well as the treatment approach versus not actively treating ECR (periodic review/watchful waiting) (Mazon et al. 2022; Mavridou et al. 2022; Patel et al. 2018). The European Society of Endodontology (ESE) and the joint position statement of the American Association of Endodontists (AAE) and American Academy of Oral and Maxillofacial Radiology (AAOMR) advocate the use of small field‐of‐view CBCT for the diagnosis and management of ECR (Patel et al. 2019; Sousa Melo et al. 2026). The use of CBCT should result in more appropriate treatment planning and therefore improved outcomes (survival) (Patel et al. 2009; Mazon et al. 2022).

With CBCT becoming more mainstream in Endodontics several studies assessing ECR treatment outcomes have included CBCT for diagnosis and/or treatment planning. In a retrospective study assessing the outcome of the treatment of 274 teeth (from an original cohort of 542) with ECR with diagnostic CBCT scans, the survival rates ranged from 84.6% (3 years), 70.3% (5 years), 42.7%, (8 years) and 28.6% (10 years), (Mavridou et al. 2022). Irinakis et al. reported a survival rate of 50% at 8 years, and DeLuca et al. had a survival rate of 70% at 1 year, however, in these studies CBCT were only used in 64.2% and 78.9%, respectively (Irinakis et al. 2022; DeLuca et al. 2023).

There remains a paucity of outcome data assessing the survival and success of the management of ECR, this is reflected in the ESE Position Statement acknowledging that ECR management options are still experimental (Bardini et al. 2023; Patel et al. 2023).

The aim of this study was to assess the survival and success rate of different treatment strategies of teeth affected with ECR when both PR and a small field of view CBCT were taken as part of the clinical assessment, treatment planning and execution of treatment.

## 2 Materials and Methods

The clinical strategy in this study was in accordance with the PROBE statement (Nagendrababu et al. 2023). This study was a retrospective, observational cohort study of 296 ECR cases seen in 5 endodontic practices; a cut‐off date to review cases was 31st January 2024.

Ethical approval was obtained from Guy’s and St Thomas’ NHS Foundation Trust Research and Development Committee (08/H0804/79, 28/09/17), and Universidad De Antioquia, Colombia 0011‐08‐2025.

Patients with ECR were identified from patient software and manual databases. The outcome of the management of ECR was assessed using the clinical records of patients who had been previously diagnosed with ECR; specific data was collected and logged onto an Excel spreadsheet (Microsoft, Richmond, WA, USA), all data was anonymized.

### 2.1 Clinical Assessment

The patient's presenting complaint (including probing, pain, cavitation, tooth discolouration, pink spot, symptoms of pulpitis and/or apical periodontitis), history of the presenting complaint and other relevant information were recorded. In some cases, there were no presenting symptoms or clinical signs. Intraoral examination was carried out using loupes and/or a dental operating microscope. When indicated, sensibility testing was carried out using both thermal and electric pulp testing.

### 2.2 Radiographic Assessment

Periapical radiographs (PRs) were obtained using a beam aiming device with a dental X‐ray machine (Nomad Pro 2 KaVo Dental Ltd., Uxbridge, UK)/digital CCD (Schick Technologies, New York, NY, USA), Heliodent X‐ray unit (Sirona, Bensheim, Germany)/PSP plates (Digora, Quakertown, PA, USA) or Carestream RVG 6200 sensors (Carestream Dental, Atlanta, Georgia, USA). Exposure parameters ranged from 60 to 70 kV, 6–7 mA. The exposure time varied between 0.13–0.26 s depending on the region (tooth/teeth) of interest.

CBCT scans were obtained with 3D Accuitomo scanner (J. Morita, Kyoto, Japan) with 4 × 4 cm field of view, with parameters of 90 kV, 5.0 mA and 17.5 s. CBCT scans were reformatted (1.5 mm slice thickness) or Carestream CS 8100 or 9000 (Carestream Dental, Atlanta, Georgia, USA) with a 4 × 5 cm field of view with exposure parameters 90 kV, 10 mA, and 20 s, reconstructed at 1.3 mm slice thickness. A CBCT scan was taken in all cases where management of ECR was planned.

The radiographic appearance (nature) of ECR was classified according to the Heithersay (1999) and Patel (3D) classification (Patel et al. 2018).

### 2.3 Management

Three different treatment approaches were carried out depending on the following criteria: symptomatology (e.g., pain, pink spot), accessibility and extent (e.g., pathological probing, predictable restorability) of ECR based on the 3D characteristics, and the presence of bone‐like tissue (Figures 1, 2, 3, 4, 5). Based on these features as well as the patient's preferences the following treatment approaches were carried out to retain the tooth in a healthy and functional state (Table 1):

- Treatment 1 (Periodic review).
- Treatment 2 (External approach).
- Treatment 3 (Internal approach).

**FIGURE 1:**
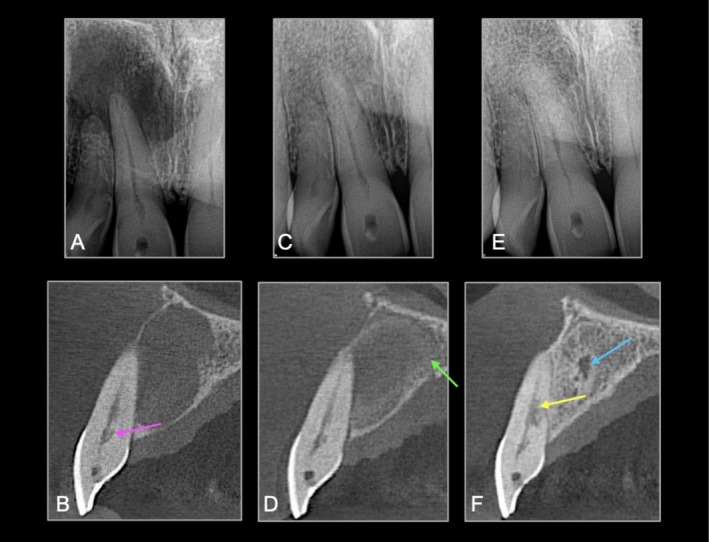
**T** **reatment option 1, Periodic review.** ECR in the upper left central incisor (21). Endodontic treatment was aborted by general dentist and referred for second opinion to an Endodontist. A diagnosis of a nasopalatine cyst was reached and referred to an Oral and Maxillo‐Facial surgeon for enucleation. (A, B) Periapical radiograph and sagittal CBCT image at consultation. There were signs of ECR in the coronal‐third of the canal (purple arrow), which the patient preferred to review on a periodic basis. (C, D) Periapical radiograph and sagittal CBCT image at 1‐year review after cyst enucleation, note the cancellous bone in fill (green arrow). (E, F) Periapical radiograph and sagittal CBCT image at 8‐year follow up reveals ECR with bone fill (yellow arrow) and significant bone infill (and scar tissue‐blue arrow).

**FIGURE 2:**
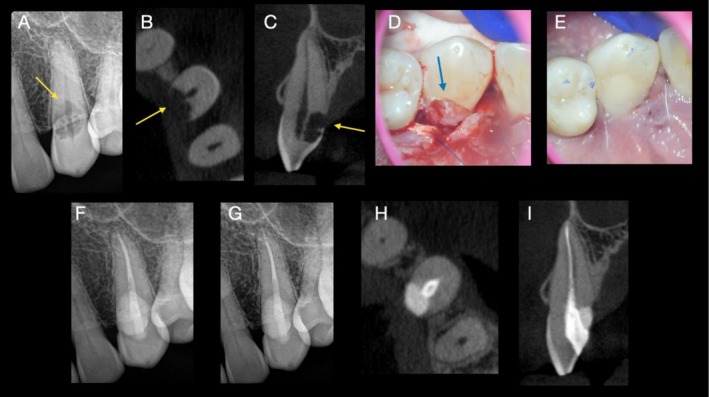
**Treatment option 2. External repair & endodontic treatment.** ECR associated with maxillary canine (23). (A) pre‐operative periapical radiograph, (B, C) pre‐operative axial and sagittal CBCT image, (D) ECR defect surgically exposed, (E) tissue repositioned and ECR defect restored, (F) post‐operative periapical radiograph, (G) 1‐year follow‐up periapical radiograph, (H, I) 1 year follow‐up axial and sagittal CBCT images.

**FIGURE 3:**
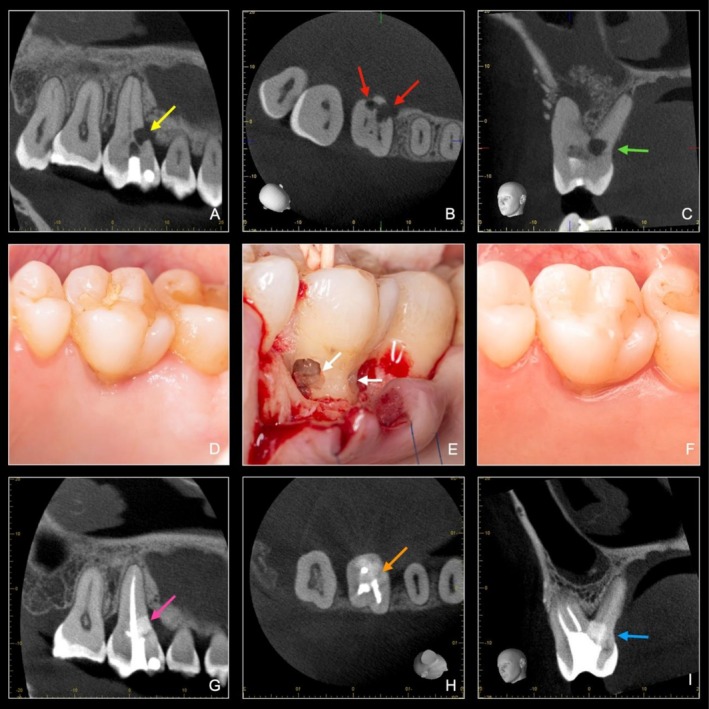
**Treatment option 2. External repair & endodontic treatment.** (A–D). Previously initiated endodontic treatment and external cervical resorption (ECR) involving the palatal root. (A) Sagittal view (yellow arrow), (B) axial view (red and orange arrows), and (C) coronal view (green arrow) from CBCT confirmed the presence of ECR. (D) Clinical examination revealed no supragingival cavitation. (E) Surgical management included flap elevation to expose the resorptive defects (white arrows). After removal of all fibroconnective tissue, the canals were protected with gutta‐percha cones to prevent obliteration during sealing of the cavities with Biodentine (Septodont, France). Endodontic treatment was then completed. (F) 2 years later, the patient presented with normal clinical appearance and (G) sagittal view (pink arrow) (H) axial view (orange arrow) and (I) coronal view (blue arrow) from CBCT revealed stable sealing of the ECR.

**FIGURE 4:**
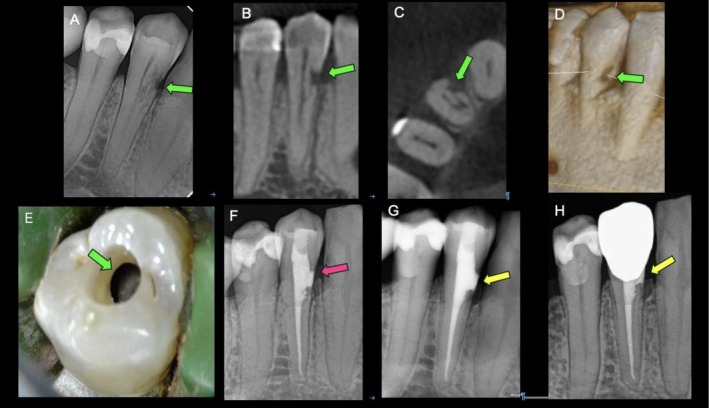
**Treatment option 3** **, Internal repair & endodontic treatment.** ECR associated with lower right first premolar (44). (A) pre‐operative periapical radiograph, (B, C) pre‐operative axial and sagittal CBCT image, (D) 3D rendered image, (E) access cavity extended to encompass the ECR lesions, (F) post‐operative periapical radiograph, (G) 1‐year follow‐up, (H) 2‐year follow‐up periapical radiographs.

**FIGURE 5:**
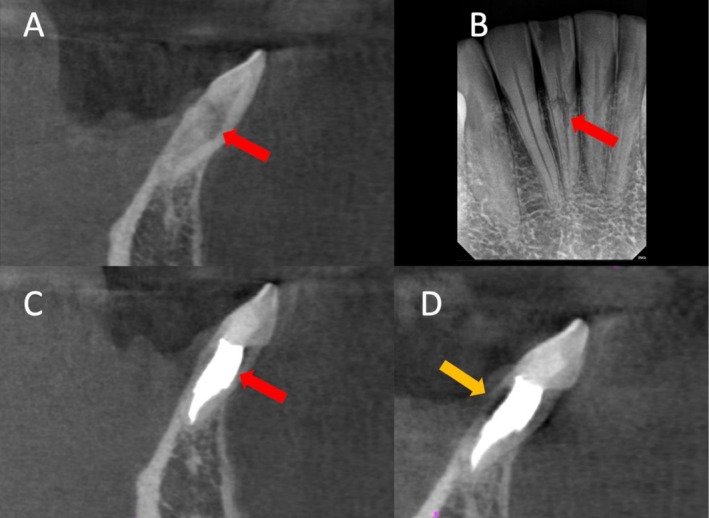
**Treatment option 3 Internal repair & endodontic treatment.** (A) Sagittal CBCT view of tooth #25 with ECR (red arrow) (B) periapical radiograph of tooth #24 with ECR (red arrow) (C) Post‐operative sagittal CBCT view showing internal‐only repair (red arrow indicates similar location in tooth to (A)). (D) 1‐year recall sagittal CBCT view showing recurrent and progressive ECR (orange arrow).

**TABLE 1:** Treatment approaches for managing (above) and treatment algorithm for external cervical resorption (ECR) (below).

Treatment type	Description
Treatment 1 *Periodic review*	Periodic reviews, asymptomatic, inaccessible, bone‐like repair
Treatment 2 *External approach*	Surgical repair with composite resin or modified glass‐ionomer cement As above with vital pulp therapy As above with endodontic treatment (single visit), +/− root resection Intentional replantation with surgical repair
Treatment 3 *Internal approach*	Endodontic treatment & internal repair of ECR defect with composite resin (+/− a base of modified glass‐ionomer cement or hydraulic cement within the debrided ECR defect)
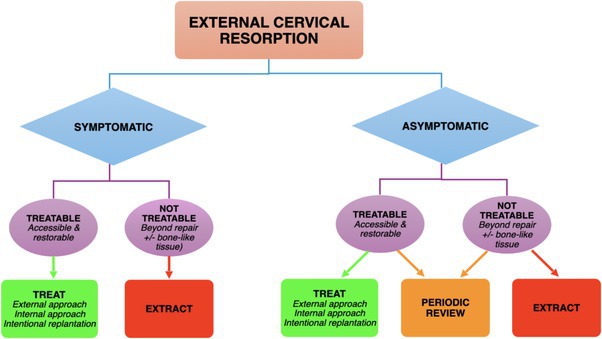	

Patients were advised to return for review appointments regardless of the treatment option chosen, however, they were advised to return sooner if they developed symptoms. Treatment 2 and 3 were carried out under local anaesthetic and managed in one or two visits. In teeth treated over two visits, interappointment calcium hydroxide (Ultracal XS, Ultradent Products Inc., South Jordan, Utah, USA) was placed into the canal space and resorptive defect (where accessible).

Treatment 1 (Periodic review) was the treatment of choice for ECR lesions which were asymptomatic, inaccessible and/or had signs of bone‐like repair. Treatment 2 involved reflecting a mucoperiosteal flap adjacent to the ECR lesion and removing the resorption tissue with an excavator, long shank burs and/or piezo tips. When possible, a dental dam was placed prior to restoring the ECR defect with a direct plastic restoration. Composite resin (G‐aenial, GC‐Europe, Bucks, UK; or Tetric Flow, Ivoclar Vivadent, Schaan, Liechtenstein) was the material of choice; however, when isolation was compromised, resin‐modified glass ionomer was used, including Geristore, (Den‐Mat Holdings, Lompoc, CA, USA), EndoSequence BC Liner (Brasseler Corp., Savannah, GA, USA), GC Fuji IX (GC America, Alsip, IL, USA), RIVA (SDI America, Itasca, IL, USA), and Vitremer (3M, Minnesota, USA). When the pulp was exposed and was deemed to be healthy (vital) at the examination stage, vital pulp therapy (VPT) was carried out following contemporary treatment guidelines (Duncan et al. 2019). A hydraulic bioceramic putty was placed over the exposed pulp tissue, such as NeoPUTTY (Avalon Biomed, Houston, TX, USA), Biodentine (Septodont, Saint‐Maur‐des‐Fossés, France), EndoSequence Root Repair Material–BC Putty (Brasseler USA, Savannah, GA, USA), or REGEN Bioactive Endo Putty (Vista Apex, Racine, WI, USA). The tooth was subsequently restored with a direct plastic restoration. Non‐resorbable sutures were used to secure the repositioned flap for 3–7 days.

In cases where the pulp was diagnosed as irreversibly inflamed, necrotic, or when elective root canal treatment was planned, the canal was accessed and temporarily occluded with a gutta‐percha point under dental dam isolation. The dental dam was then removed when necessary, the ECR lesion was excavated and managed as previously described, and the dam was subsequently repositioned to allow completion of the endodontic treatment. When completed over two visits, calcium hydroxide intra‐canal medicament was placed into the canal space and the resorptive defect, where accessible. The root canal system was irrigated with 3% sodium hypochlorite, the irrigant was replenished every 1–2 min after which it was immediately agitated with an appropriately selected gutta percha point extending to 2 mm short of the working length for approximately 30 s, Laser‐assisted irrigation (LAI) or passive ultrasonic activation was implemented. The root canals were instrumented with a combination of stainless steel and nickel‐titanium files which were contemporary at the time of treatment. The root canals were then irrigated with ethylenediaminetetraacetic acid (EDTA) (ENDO‐Solution (PPH Cerkamed)) followed by a final irrigation with sodium hypochlorite. The canals were then obturated with gutta percha and hydraulic cement (Endosequence BC Sealer (Hiflow, Brasseler Corp., Savannah, GA, USA)); NeoSealer (Avalon Biomed, Houston, TX, USA) or REGEN Bioactive Sealer (Vista Apex Solutions, Racine, WI, USA). The access cavity was sealed immediately with a composite resin. Intentional replantation was carried out when indicated, the extra‐oral time was less than 10 min, and the ECR defect was restored with resin‐modified glass‐ionomer cement. The tooth was splinted in place for 2 weeks, and single visit endodontic treatment was carried out prior to removal of the splint (Plotino et al. 2021).

Treatment 3 was indicated for cases which were not readily accessible to treatment 2 (e.g., interproximal ECR in multi‐root treated and/or > 270°+ circumferential spread). The majority of these cases were treated in a single visit as described above. When resorption was more extensive or bleeding uncontrollable a 2‐visit approach was necessary, and the root canal system was dressed with a calcium hydroxide intracanal medicament. In treatment 2 and 3 a microbrush soaked with sodium hypochlorite or 90% trichloroacetic acid (TCA) was scrubbed into the dentine after ECR defect had been excavated.

Patients with symptomatic, untreatable (i. e, inaccessible and/or too extensive to predictably manage) ECR lesions were advised to have the affected tooth extracted; these patients were not included in this study.

### 2.4 Follow‐Up

Reviews were carried out on an annual basis. Patient's symptoms were evaluated, followed by a detailed clinical examination that included periodontal probing of the affected tooth per the initial clinical examination.

Three main outcome criteria were defined:

- Failure: loss of the tooth due to pain, resorption progression, vertical root fracture, restorative failure and/or periodontal problems.
- Success: Asymptomatic, clinically healthy and radiographic signs of healing or absence of a periapical radiolucency.
- Survival: Asymptomatic and functional (retention) including teeth in which ECR recurred.

### 2.5 Statistical Analysis

All statistical analyses were performed using RStudio software (version 2025.05.0 + 496, Posit Software, PBC, Boston, MA, USA) with the *survival* and *coxme* packages. The primary outcome was tooth survival, defined as the time from diagnosis or treatment to extraction. Recall time was recorded in years. Teeth that had not been extracted by the end of the follow‐up period were censored at their last available review, as the extraction event had not occurred during the observation period.

Descriptive statistics were used to summarize patient demographics, tooth characteristics, ECR classification, and treatment types. The recall rate was calculated at specific time intervals (1, 2, 5, 7, and 10 years) based on the number of teeth with clinical and/or radiographic follow‐up available at each time point.

Kaplan–Meier survival curves were generated to estimate cumulative tooth survival and compared across groups using the log‐rank test. Survival probabilities were reported at 1, 2, 5, 7, and 10 years. To assess the impact of individual variables on tooth survival, univariate Cox proportional hazards models with mixed effects were fitted using the (coxme) function, accounting for clustering by patient ID as a random effect. Variables included in univariate models were age, gender, tooth type, anterior/posterior location, Heithersay classification, Patel classification (height, circumferential spread, and depth), and treatment type. Hazard ratios (HR) with 95% confidence intervals (CI) and *p*‐values were reported. A multivariate Cox mixed‐effects model was then constructed including variables that were clinically relevant and/or statistically significant in univariate analysis to assess their independent effect on tooth survival.

All statistical tests were two‐sided, and a *p*‐value < 0.05 was considered statistically significant.

## 3 Results

A total of 305 patients with 368 teeth affected by external cervical resorption (ECR) were initially identified. After excluding 72 patients (and their 72 teeth) due to immediate extraction, follow‐up of less than 1 year, or lack of follow‐up, the final analysis included 233 patients with 296 teeth. Of the patients, 34 (14.6%) had multiple teeth involved, accounting for 97 teeth (32.7%) (Table 2). The distribution of patients with multiple teeth was higher in males (*n* = 21) than females (*n* = 13).

**TABLE 2:** Distribution of patients with multiple teeth affected by external cervical resorption (ECR).

*n* of teeth	*n* of patients with multiple teeth	Total *n* of teeth
2 teeth	24	48
3 teeth	4	12
4 teeth	3	12
5 teeth	1	5
6 teeth	1	6
14 teeth	1	14
Total	34	97

The sample consisted of 106 males (45.5%) contributing 152 teeth (51.4%), and 127 females (54.5%) with 144 teeth (48.6%). The overall, mean age (±SD) was 51.7 years (±16.7). The mean age of males and females was 47.5 (SD±17.8) and 55.3 years (SD±14.9), respectively (Table 3). The age group most represented was 61–70 years (25.8%), followed by 51–60 years (20.2%).

**TABLE 3:** Age distribution of patients with external cervical resorption ECR.

Age category	Age group	*n*	%	Male	%	Female	%
1	Less than 30	27	11.6%	20	18.9%	7	5.5%
2	31–40	35	15.0%	17	16.0%	18	14.2%
3	41–50	39	16.7%	18	17.0%	21	16.5%
4	51–60	47	20.2%	21	19.8%	26	20.5%
5	61–70	60	25.8%	22	20.8%	38	29.9%
6	Above 70	25	10.7%	8	7.5%	17	13.4%
	Total	233	100.0%	106	100.0%	127	100.0%

### 3.1 Teeth Distributio*n*

The teeth distribution included incisors 45.3% (*n* = 134 [79 maxillary/55 mandibular]), canines 20.3% (*n* = 60 [36 maxillary/24 mandibular]), premolars 13.2% (*n* = 39 [17 maxillary/22 mandibular]), and molars 21.3% (*n* = 63 [23 maxillary/40 mandibular]), with a nearly equal distribution between maxillary (52.4%) and mandibular (47.6%) locations (Figure 6).

**FIGURE 6:**
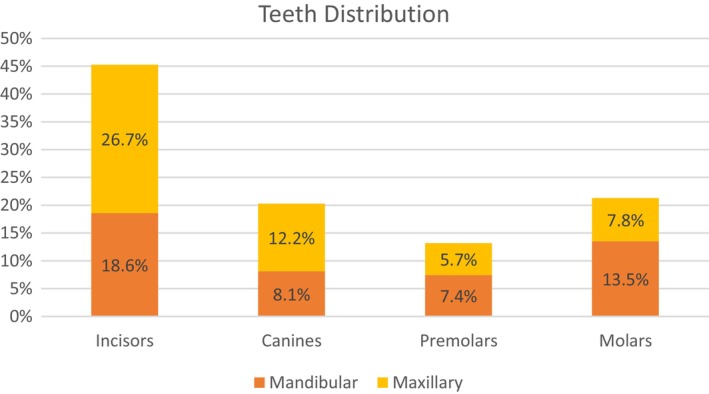
Distribution of teeth by type (incisors, canines, premolars, molars) in the maxilla and mandible. The figure illustrates the frequency and proportion of each tooth type affected in both jaws.

### 3.2 Classification of ECR Lesions

The distribution of ECR lesions based on the Heithersay (2D) classification (Class I–IV) and the Patel (3D) classification (height, circumferential spread, and depth) is summarized in the corresponding Table 4 and Figures 7, 8.

**TABLE 4:** Distribution of external cervical resorption (ECR) according to the Heithersay (2D) classification.

Heithersay classification	*n*	%
CL I	63	21.3%
CL II	78	26.4%
CL III	98	33.1%
CL IV	57	19.3%
Total	296	100.0%

**FIGURE 7:**
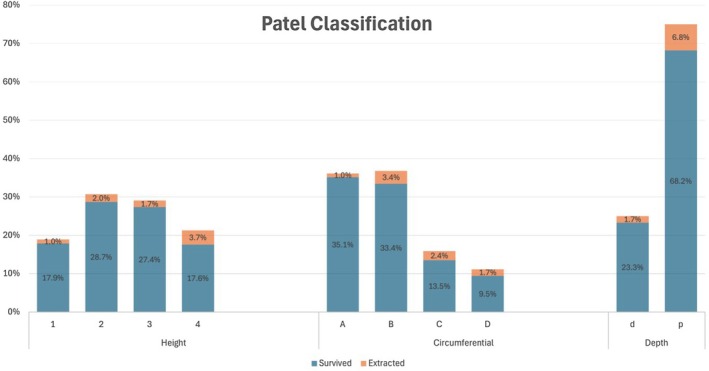
Distribution of ECR based on Patel classification, and the survival/extraction rate.

**FIGURE 8:**
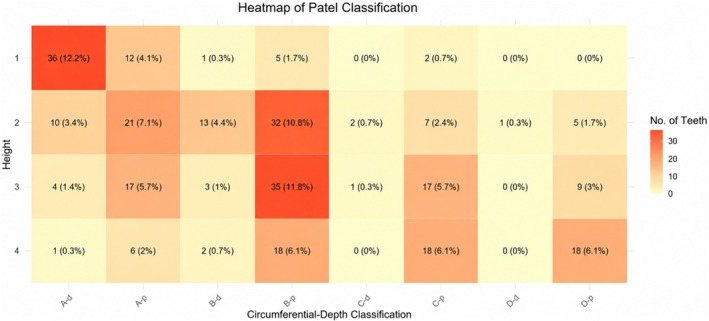
Heatmap illustrating the percentage distribution of ECR according to the Patel classification system.

### 3.3 Observational Period

The follow‐up duration ranged from 12 to 123 months (Table 5), with a mean observational period of 33.4 months (approximately 2.9 years). The majority of teeth were followed for less than 5 years (78.4%), with 51 teeth (17.2%) followed between 5 and 7 years and only 13 teeth (4.4%) contributing data beyond 7 years.

**TABLE 5:** Distribution of follow‐up duration for teeth with external cervical resorption (ECR).

Recall time (years)	*n* of teeth	%
1 year	27	9.1%
1‐2 years	100	33.8%
2‐5 years	105	35.5%
5‐7 years	51	17.2%
7+ years	13	4.4%

### 3.4 Treatment Type and Survival Rates

The 296 teeth were managed by periodic reviews (treatment 1) in 37.5% (*n* = 111) cases, external approach (treatment 2) in 33.8% (*n* = 100) cases (external repair with or without vital pulp therapy, external repair combined with endodontic treatment ± root resection, or external repair with intentional replantation), and internal approach (treatment 3) in 28.7% (*n* = 85) cases (internal repair with endodontic treatment, or endodontic treatment alone).

The estimated cumulative survival rates were 93.6%, 90.2%, and 59.1% for treatment 1, treatment 2, and treatment 3, respectively, based on Kaplan–Meier analysis, with follow‐up extending to a maximum of 10 years. The majority of observations were concentrated within the first 5–7 years of follow‐up. The distribution of treatment modalities according to 3D lesion severity, as defined by the Patel classification, is presented in Table 6.

**TABLE 6:** Distribution of treatment type according to the Patel classification.

3D Domain	Severity level	Periodic review *n* (%)	External repair *n* (%)	Internal Repair *n* (%)	Total
Height	1	21 (37.5)	19 (33.9)	16 (28.6)	56
	2	16 (17.6)	49 (53.8)	26 (28.6)	91
	3	44 (51.2)	23 (26.7)	19 (22.1)	86
	4	30 (47.6)	9 (14.3)	24 (38.1)	63
Circumferential	1	36 (33.6)	41 (38.3)	30 (28.0)	107
	2	34 (31.2)	47 (43.1)	28 (25.7)	109
	3	25 (53.2)	8 (17.0)	14 (29.8)	47
	4	16 (48.5)	4 (12.1)	13 (39.4)	33
Depth	1	30 (40.5)	30 (40.5)	14 (18.9)	74
	2	81 (36.5)	70 (31.5)	71 (32.0)	222

### 3.5 Predictors of Tooth Survival: Univariate Cox Regression Analysis

#### 3.5.1 Gender

Univariate Cox proportional hazards analysis revealed that females had a slightly higher hazard of extraction compared to males (HR = 1.07; 95% CI: 0.42–2.72; *p* = 0.897), but this was not statistically significant.

#### 3.5.2 Age Group

In the univariate analysis, none of the age groups demonstrated a statistically significant association with tooth survival when compared to the youngest group (Age Group 1, reference, < 30 years old). Hazard ratios varied across categories, with some age groups (e.g., Age Groups 2, 3, and 6) showing HRs below 1, suggesting a potential trend toward improved survival, while others (e.g., Age Group 5 (61–70 years old)) had HRs above 1. However, these associations were not statistically significant (*p* > 0.05 for all).

#### 3.5.3 Tooth Group

Univariate analysis revealed that posterior teeth demonstrated a lower risk of extraction compared to anterior teeth (HR = 0.80; 95% CI: 0.30–2.19), although not statistically significant (*p* > 0.05).

#### 3.5.4 Tooth Location

In the univariate analysis, mandibular teeth showed a lower hazard (risk) of extraction compared to maxillary teeth (HR = 0.63; 95% CI: 0.25–1.61); however, the difference was not statistically significant (*p* > 0.05).

The distribution of external cervical resorption (ECR) lesions according to the 3D classification system (height, circumferential spread, and depth) for anterior and posterior teeth is summarized in Table 7.

**TABLE 7:** Patel classification of external cervical resorption (ECR) lesions by height (1–4), circumferential spread (A–D), and depth (d, p), with percentages for anterior and posterior teeth.

Patel classification						
Height	*n*	%	Anterior	%	Posterior	%
1	56	18.9%	30	10.1%	26	8.8%
2	91	30.7%	59	19.9%	32	10.8%
3	86	29.1%	59	19.9%	27	9.1%
4	63	21.3%	46	15.5%	17	5.7%
Total	296	100.0%				

Circumferential	*n*	%	Anterior	%	Posterior	%
A	107	36.1%	68	23.0%	39	13.2%
B	109	36.8%	74	25.0%	35	11.8%
C	47	15.9%	26	8.8%	21	7.1%
D	33	11.1%	26	8.8%	7	2.4%
Total	296	100.0%				

Depth	*n*	%	Anterior	%	Posterior	%
d	74	25.0%	46	15.5%	28	9.5%
p	222	75.0%	148	50.0%	74	25.0%
Total	296	100.0%				

#### 3.5.5 Heithersay Classification and Tooth Survival

With univariate analysis, tooth survival was not related to the severity (class I‐IV) of ECR based on Heithersay classification. Compared to Heithersay class 1 (reference group), the hazard ratios for Heithersay class 2, 3, and 4 were 0.55 (95% CI: 0.10–3.01; *p* = 0.49), 1.26 (95% CI: 0.32–4.96; *p* = 0.73), and 2.93 (95% CI: 0.69–12.48; *p* = 0.15), respectively. While class 3 and 4 showed elevated hazard ratios indicating a potential increased risk of extraction, these findings did not reach statistical significance. Overall, the Heithersay classification did not demonstrate a significant impact on survival outcomes in this unadjusted analysis.

#### 3.5.6 Patel Classification and Tooth Survival

The association between 3D classification and tooth survival was evaluated through both individual categories and grouped comparisons.

Height‐ Using height 1 (supracrestal) as the reference, height 2 (subcrestal) was associated with a modestly increased risk of extraction (HR = 1.37; 95% CI: 0.28–6.58; *p* = 0.697), as was height 3 (HR = 1.36; 95% CI: 0.28–6.65; *p* = 0.704); however, neither association reached statistical significance. Height 4 demonstrated a statistically significant association with increased extraction hazard (HR = 5.40; 95% CI: 1.20–24.36; *p* = 0.029).

When height 1 and 2 were grouped as ‘Minor’ and height 3 and 4 as ‘Major’, the latter group demonstrated a higher hazard of extraction compared to the ‘minor’ group (HR = 2.21; 95% CI: 0.85–5.69), although this association did not reach statistical significance (*p* = 0.102).

Circumferential spread—The impact of circumferential spread of ECR (A–D) on tooth survival was analysed using individual types and grouped categories. Using circumferential spread group A (< 90°) as the reference, group B (> 90°–< 180°) demonstrated a higher hazard of extraction (HR = 3.76, 95% CI: 0.93–15.23, *p* = 0.063), although not statistically significant. Group C (> 180°–< 270°) showed a statistically significant association with increased extraction hazard (HR = 7.86; 95% CI: 1.80–34.37; *p* = 0.006). Group D (> 270°) also showed an increased risk (HR = 3.43; 95% CI: 0.65–18.02; *p* = 0.144), but the association was not statistically significant.

When circumferential types were grouped into ‘Low’ (group A and B) versus ‘High’ (group C and D), the High group demonstrated a more than twofold increased hazard of extraction (HR = 2.42; 95% CI: 0.99–5.94), with a borderline significant *p*‐value (*p* = 0.054). This trend suggests that teeth with more extensive circumferential involvement (> 180°+) are at greater risk of extraction.

Proximity to root canal‐Group p (Probable or actual pulp involvement) resulted in a higher hazard of extraction compared with group d (Lesion confined to dentine) (HR = 1.52; 95% CI: 0.49–4.73), although this association did not reach statistical significance (*p* > 0.05).

### 3.6 Influence of Treatment Type on Tooth Survival

With univariate analysis, treatment type demonstrated variability in association with tooth survival. Using treatment 1 (periodic review) as the reference category, treatment 2 (external repair) was associated with a nearly twofold higher hazard of extraction (HR = 1.98; 95% CI: 0.51–7.67; *p* = 0.322), although this association did not reach statistical significance. Treatment 3 (internal repair) was associated with a statistically significant fivefold higher hazard of extraction (HR = 5.52; 95% CI: 1.65–18.43; *p* < 0.01).

### 3.7 Predictors of Tooth Survival: Multivariate Cox Mixed‐Effects Model

A multivariate Cox mixed‐effects model was generated to explore the combined association of demographic, clinical, and radiographic variables on tooth survival. The model included age groups, gender, tooth type, tooth location, 2‐ and 3‐ dimensional classifications of ECR, and treatment type as fixed effects, with patient ID modelled as a random effect. Given the limited number of failure events, these analyses were considered exploratory.

Among demographic variables, no age group or gender category showed statistically significant associations with extraction risk. Tooth location demonstrated a significant association with survival, with mandibular teeth showing a lower hazard of extraction compared with maxillary teeth (HR = 0.31; 95% CI: 0.10–0.90; *p* = 0.032).

Internal repair (treatment 3) remained associated with a higher hazard (sevenfold) of extraction compared with the treatment 1 (periodic review) reference group (HR = 7.23; 95% CI: 1.91–27.41; *p* = 0.004), whereas treatment 2 (external repair) demonstrated a slightly higher (approximately threefold) hazard non‐significant association (HR = 2.97; 95% CI: 0.66–13.42; *p* = 0.158).

Within the 3D classification variables, circumferential extent group C (> 180°–< 270°) demonstrated an association with a higher hazard of extraction (HR = 12.9; 95% CI: 1.81–93.10; *p* = 0.011). Height category 2 also showed a borderline association with extraction hazard (HR = 6.62; 95% CI: 1.03–42.6; *p* = 0.047). Other extent and depth categories did not demonstrate statistically significant associations.

### 3.8 Cumulative Survival Rates

The estimated survival probability at 1 and 2 years was 95.5% (95% CI: 0.93–0.98) and 93.3% (95% CI: 0.90–0.97), respectively. At 5 and 7 years, the survival rate was 85.7% (95% CI: 0.80–0.92) and 80.5% (95% CI: 0.72–0.90), respectively. Beyond 7 years, the survival curve demonstrated minimal additional change; however, the number of teeth remaining at risk was very small at the longest time points (*n* = 3), limiting the precision of these estimates. Overall, despite a gradual decrease over time, the majority of teeth remained intact through the 7‐year follow‐up period (Table 8, Figure 9). During the follow‐up period, 25 teeth failed, with 13 failures attributed to recurrence of ECR and 12 to non‐restorability.

**TABLE 8:** Cumulative survival rates of teeth with ECR at 1, 2, 5, 7 and 10 years.

Time Point	Teeth at risk	Events	Cumulative survival	95% Confidence interval
1 Year	269	12	95.5%	93.1–98.0
2 Years	169	4	93.3%	90.1–96.6
5 Years	64	7	85.7%	79.6–92.3
7 Years	14	2	80.5%	71.9–90.3
10 Years	3	0	80.5%	71.9–90.3

**FIGURE 9:**
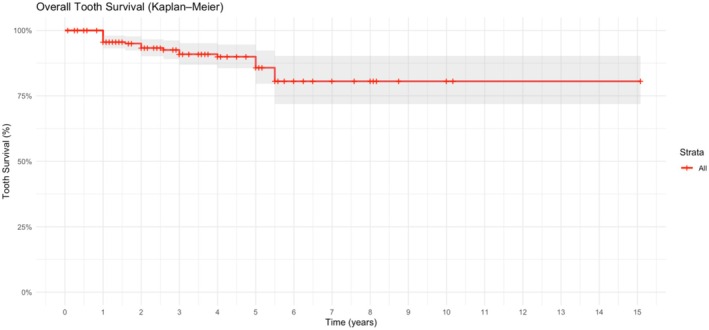
Kaplan–Meier survival curve showing the cumulative survival probability of teeth with external cervical resorption (ECR) over time.

Kaplan–Meier survival curves demonstrated differences in estimated tooth survival outcomes observed across the three treatment categories over the observation period. The periodic review group (treatment 1; *n* = 111 at baseline) demonstrated higher estimated survival probabilities across the follow‐up period, with a small number of teeth remaining at risk at later time points. The external repair group (treatment 2) demonstrated slightly lower estimated survival probabilities compared with periodic review, with relatively similar trajectories during the first 5 years of follow‐up with an estimated survival probability of approximately 90% at 5 years.

In contrast, ‘Internal repair’ (treatment 3) demonstrated lower estimated survival probabilities, with a steeper decline observed during the first 5 years and continued decline thereafter; however, the number of teeth contributing data at later time points was limited. The accompanying risk table illustrates the decreasing numbers at risk over time. Differences between survival curves were statistically significant based on the log‐rank test (*p* = 0.001) (Figure 10).

**FIGURE 10:**
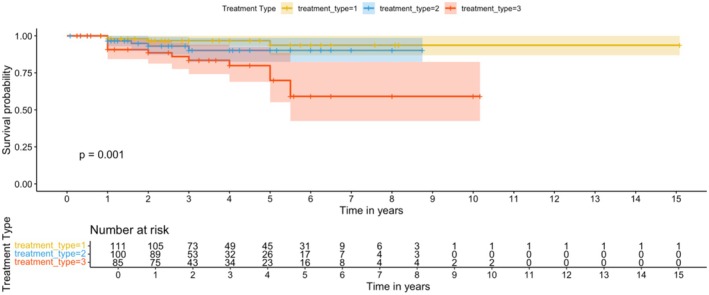
Kaplan–Meier survival curves showing differences in estimated tooth survival across treatment types over time.

### 3.9 Cumulative Success Rates

At 1 year, the cumulative tooth success rate was 94.4% (95% CI: 0.92–0.97). At 2 years, success was 84.5% (95% CI: 0.80–0.90), decreasing to 62.7% (95% CI: 0.55–0.72) at 5 years and 50.9% (95% CI: 0.41–0.63) at 7 years. The estimated success rate at 10 years was 45.9% (95% CI: 0.35–0.62); however, the number of teeth contributing data at later time points was limited. The Kaplan–Meier success curve (Figure 11) illustrates the decline in tooth success probability over time.

**FIGURE 11:**
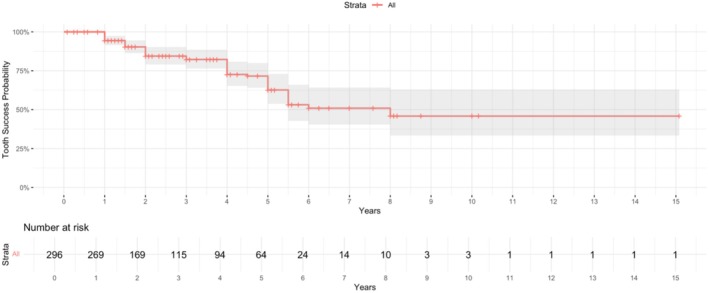
Kaplan–Meier survival curve showing the cumulative success probability of teeth with external cervical resorption (ECR) over time.

## 4 Discussion

The principal aim of this retrospective cohort study was to evaluate the long‐term survival and success of teeth affected by ECR and managed using different approaches according to symptomatology and nature of the resorption lesion within specialist endodontic practice. A secondary aim was to explore associations between demographic, clinical, and radiographic variables and treatment outcomes.

In the present cohort, overall tooth survival was 91.6% (271/296 teeth) based on a mean follow‐up period of 2.9 years; with 25 teeth (8.4%) extracted during follow‐up. The short‐term survival rate over 1 and 2 years was 95.5% and 93.3%, respectively. The survival rate reduced over 5 and 7 years to 85.7% and 80.5%, respectively. These results are in the same order of magnitude as those reported by Mavridou et al. who observed survival rates of between 84.6% and 70.3% at 3 and 5 years, respectively, in a cohort of 274 teeth (Mavridou et al. 2022).

The highest survival rates in this study were observed in teeth managed with periodic review (treatment 1), with survival rates of 98.1% at 1 year, 96.8% at 2 years, and 93.6% at 5 to 7+ years, highlighting the favourable long‐term outcomes associated with periodic monitoring when indicated. In contrast, Mavridou et al. (2022) reported a survival rate of 70.3% with a mean follow‐up of 5 years for periodic reviews (treatment 1). This treatment approach is recommended when there are signs of bone‐like repair, no symptoms and/or probing is not pathological in nature (Mavridou et al. 2022; Patel et al. 2023). In the present cohort, periodic review (treatment 1) was the most common management strategy, this is consistent with findings reported by Mavridou et al. (2022); Irinakis et al. (2022).

DeLuca reported periodic reviews (treatment 1) had a 100% survival rate compared to actively managed (treatment 2 and 3), which had a survival rate of 81.5% when reviewed at 1 year (DeLuca 2025). Irinakis et al. (2022) observed that the cumulative failure probability reached 50% by approximately 8 years, implying a progressively increasing hazard over time. While the hazard associated with ECR active management versus no active management, and with internal versus external repair in their study appeared similar in the early years, teeth that did not receive root canal treatment, and those with ECR repair without endodontic treatment experienced noticeably higher failure hazards throughout the follow‐up period. In these studies, diagnostic CBCT imaging was performed in 78.9% of cases in the DeLuca cohort (DeLuca et al. 2023) and in 64.2% of cases in the Irinakis cohort (Irinakis et al. 2022). It has been demonstrated that PRs may underestimate the extent of ECR when compared with CBCT, Villefrance et al. (2022) reported that lesion severity was judged to be greater in 38% of cases and less severe in 3% of cases when CBCT assessment was compared with PR. These findings highlight the importance of three‐dimensional imaging for accurate lesion characterization and treatment planning; however, the present study was not designed to evaluate whether CBCT influenced clinical outcomes.

CBCT was used for every case in the present study; this strengthened diagnostic accuracy as well as appreciation of the nature of ECR, thus enhancing treatment planning as well as patient consent. CBCT has been shown to improve detection and three‐dimensional assessment of ECR and to influence treatment planning when compared with periapical radiography (Patel et al. 2009; Rodriguez et al. 2017; Rodríguez Mazón et al. 2022). However, the present study was not designed to evaluate whether CBCT use influences clinical outcomes, and no causal inference regarding survival can be made.

In addition, several published studies have included relatively small sample sizes, including 72 teeth in the DeLuca cohort (DeLuca et al. 2023) and 89 teeth in the Irinakis cohort (Irinakis et al. 2022), which may limit generalisability and statistical power. Smaller cohorts tend to reduce the ability to detect meaningful differences between treatment strategies and may not fully capture the variability observed in larger and more heterogeneous clinical populations (Button et al. 2013).

In the present study, the cumulative tooth success rate was relatively high in the short‐term and gradually declined with longer follow‐up. At 1 year, success was 94.4%, decreasing to 84.5% at 2 years, 62.7% at 5 years, and 50.9% at 7 years. Compared with Mavridou et al. (2022), who reported markedly lower success rates of 49.8% at 3 years, 41.3% at 5 years, and 17.5% at 8 years, our findings demonstrate more favourable outcomes, particularly within the first 5 years. Jebril et al. (2020) reported a success rate of 79% at a mean follow‐up of 20 months. While the assessment of long‐term prognosis remains challenging, the present findings indicate comparatively higher medium‐term success within this cohort. The differences in results between studies may reflect variations in case selection, lesion characteristics, operator experience, outcome definitions, and follow‐up protocols. In the present study, treatments were performed by experienced endodontists in specialist practice reflecting real world endodontics, whereas other cohorts included a combination of specialists and postgraduate trainees managed in teaching institutions/schools, which may have influenced reported outcomes.

In the present study, the recall rate was 90.9% at 1 year (269 teeth), decreasing to 57.1% at 2 years (169 teeth), 21.6% at 5 years (64 teeth), 4.7% at 7 years (14 teeth), and only 1.0% at 10 years (3 teeth). This trend aligns with the findings of Mavridou et al. (2022), who also reported a markedly reduced number of recalled teeth after 8 years, underscoring a common challenge in long‐term retrospective follow‐up studies. Although Irinakis et al. (2022) reported higher follow‐up rates (90.8% of teeth), their smaller sample size may have enabled better retention. Comparison with previous studies assessing ECR outcomes was challenging, as recall rates were not consistently reported. It has been reported that a recall rate of 80%+ is desirable to reduce bias and improve the accuracy of the results, however, the challenges in achieving high recall rates are recognized (da Silva et al. 2019, Sprague et al. 2003; Ross et al. 2009). Typically, the reasons for patients being unwilling to attend recalls are (in)direct costs (e.g., transport, time away from home or work) and transient nature of workforce in large cities (Friedman et al. 2003; Sprague et al. 2003; Ng et al. 2011). As the follow‐up period increases, the recall rate generally declines, which may reduce the validity and reliability of long‐term outcome assessments.

In total, 37.5% of teeth (*n* = 111) were periodically reviewed (treatment 1). An external approach (treatment 2) was performed in 33.8% of cases (*n* = 100), which included the following subgroups: external repair with root canal treatment (22.0%), external repair only (8.4%), external repair combined with vital pulp therapy (1.4%), and combined external and internal repair with root canal treatment and root resection (2%). An internal approach (treatment 3) was performed in 28.7% of cases (*n* = 85). In a similar study, the breakdown of treatment 1, 2, and 3 was 50.7%, 32.5%, and 16.8%, respectively (Mavridou et al. 2022). Trichloroacetic acid (TCA) was used in less than 10% of cases, while sodium hypochlorite was routinely used; therefore, no statistical analysis was performed on this variable.

The ‘periodic review’ (treatment 1) option yielded the highest estimated cumulative survival rate, 93.6% up to 10 years; this may be due to this group typically including ECR with bone‐like tissue indicating repair of the resorbed cavity. Furthermore, attempting to actively manage some of the teeth may have resulted in inadvertent removal of sound dentine weakening the tooth further and resulting in premature demise. In univariate analysis, the active treatment options, ‘external repair’ (treatment 2) and ‘internal repair’ (treatment 3) options had a two‐times and five‐times extraction rate, respectively. The more favourable results with an external approach (treatment 2) may be due to a more dentine‐conserving approach compared to the internal approach (treatment 3), which would result in more dental hard tissue removal to access the ECR lesions. Nevertheless, the survival estimates remained relatively high for both approaches within the available follow‐up, with approximately 90.2% survival reported at 7 years for external repair and 59.1% at later time points for internal repair, although the number of teeth contributing data at extended follow‐up was limited. Consequently, differences in survival across treatment groups should be interpreted in the context of baseline lesion characteristics and clinical decision‐making rather than as reflecting intrinsic treatment effectiveness.

Although treatment allocation reflected lesion characteristics and clinical feasibility, all three treatment categories were represented across the full spectrum of 3D severity domains (Table 6), reducing the likelihood of complete channelling of more severe lesions into a single treatment group; nevertheless, residual confounding by indication cannot be excluded.

Comparable trends have been reported in previous studies. DeLuca et al. observed no failures among teeth managed by periodic review (treatment 1) and an 81.5% survival rate among actively treated teeth (treatment 2/3) at 1‐year follow‐up (DeLuca et al. 2023). Periodic review (treatment 1) was also reported to have the highest survival rate in Mavridou's et al. retrospective outcome study of 274 ECR lesions with up to a 7‐year follow‐up, however, a comparison of the ‘external’ and ‘internal’ approach was not possible as they had included a separate treatment option ‘combination (internal/external approach)’ which included teeth which were externally repaired and had an endodontic treatment (Mavridou et al. 2022); (Irinakis et al. 2022) also found a higher survival rate for external repair. Survival estimates beyond 7 years should be interpreted cautiously and regarded as descriptive due to the small number of teeth involved and low failure rate.

With the Patel classification there was an increased hazard of extraction when the height was > 3, and when the circumferential spread was > 180°, this may reflect increased loss of dentine due to ECR, coupled with an increased likelihood of an internal treatment approach resulting in inadvertent removal of sound dentine to access the ECR resulting in weakening of the pericervical dentine.

The higher estimated survival rate of posterior compared to anterior teeth may be due to relatively more remaining volume of (pericervical) dentine and therefore a more favourable (increased) dentine to pulp space/ECR ratio in posterior compared to anterior teeth. This was reflected in the anterior tooth group being more commonly affected with advanced (higher) ECR classifications than posterior teeth.

The treatment categories followed the ESE Position Statement on Root Resorption (Patel et al. 2023). The most frequent treatment approaches to least frequent were periodic review, external repair and then internal repair, similar trends were reported in other retrospective ECR outcome studies (Mavridou et al. 2022; Irinakis et al. 2022). For statistical purposes, two cases of intentional replantation were included in the external repair group, given their surgical nature, treatment approach and the limited sample size, which did not allow for separate analysis. A total of 72 teeth were not assigned to any treatment group as they were either immediately extracted following clinical consultation due to being symptomatic and untreatable or were lost to follow‐up.

The clinicians who contributed cases to this study were all experienced endodontists with at least 10 years' experience in endodontic practice as well as teaching at postgraduate level. The clinicians all worked in group practices and routinely discussed complex cases, including ECR cases in regular multi‐disciplinary treatment planning sessions, following a similar methodology described in previous retrospective ECR outcome studies (DeLuca et al. 2023; Irinakis et al. 2022; Mavridou et al. 2022). A limitation of this study was that inter‐ and intra‐examiner reliability assessments were not performed due to being retrospective in nature. However, the high survival rates in the present study validate the reliability of the clinician's diagnosis, treatment planning and execution of treatment of ECR. Lesion assessment and treatment planning were guided by clinical case‐based, multidisciplinary sessions; however, some degree of subjective interpretation cannot be excluded. The present study represented real world endodontics, where clinicians reflect and discuss cases among colleagues within their clinical practice. Future prospective clinical studies would need to include inter‐ and intra‐examiner reliability to ensure consistency and reproducibility. A limitation of the existing body of literature is that they are generally based on smaller sample sizes and/or are conducted in an academic setting which may not accurately represent the setting of specialist practice where the majority of this type of treatment is performed.

In this study, the main reasons for failure were recurrence of ECR and non‐restorability, which aligns with previous reports attributing failures to the continued progression of resorption (Jebril et al., 2020; Mavridou et al. 2022) and loss of restorability (Irinakis et al. 2022). However, unlike Mavridou et al. (2022), who identified vertical root fractures as a major cause of long‐term failures, this was not observed in our cohort.

A notable discrepancy was observed between the Heithersay (2D) and Patel (3D) classifications, with a mismatch in 32.5% of anterior teeth (*n* = 63/194) and an even higher discrepancy of 39.2% in posterior teeth (*n* = 40/102). This difference suggests that reliance on the Heithersay classification may be particularly limiting, especially when evaluating posterior teeth, where the complex anatomy may not be fully appreciated using 2D imaging due to anatomical noise and geometry. These findings further highlight the clinical relevance of using a 3D‐based classification system and raise concerns about the validity of conclusions drawn from studies that rely solely on 2D‐based classification methods.

Tooth survival (functional retention) was used as the primary measure for outcome as it is the most critical patient‐reported outcome measure and is advocated as the ‘most critical outcome measure’ by the ESE (Duncan et al. 2021, ESE S3 guidelines). Tooth survival is also relevant for patients when considering the outcome of different treatment options (e.g., endodontic versus extraction/dental implant treatment options) (Fransson and Dawson 2023).

The advantage of multi‐centre retrospective outcome studies is an increased sample size which enhances statistical power as well as assessing a more diverse patient population thus yielding more robust and generalizable results compared to single‐centre studies, especially when carried out in clinical practice. These types of studies also minimize financial outlay. Ideally, future studies on this topic would be prospective in nature to minimize bias; however, these types of studies are expensive to run, have longer time frames as well as patient attrition.

## 5 Conclusion

In this multi‐centre retrospective cohort, higher estimated survival was observed among teeth managed by periodic review (treatment 1). Periodic review was associated with the highest estimated survival within the observed follow‐up; however, this may be in part due to the presence of bone‐like tissue indicating natural repair of the resorbed cavity. Active interventions showed higher extraction rates, with external repair (treatment 2) demonstrating more favourable estimated survival than internal repair (treatment 3) within the observed follow‐up period. Despite this, both active approaches maintained relatively high survival rates, supporting their use when intervention is indicated.

The treatment option chosen was influenced by the severity, accessibility, and biological features of ECR. Therefore, differences in survival likely reflect case selection rather than the intrinsic superiority of one treatment option over another.

Estimated cumulative survival within the available follow‐up was 93.6% for periodic review, 90.2% for external repair, and 59.1% for internal repair. In multivariable analysis, internal repair remained associated with a higher hazard of extraction (sevenfold), whereas external repair demonstrated a threefold hazard of extraction (non‐significant). However, extrapolating these findings to long‐term clinical case success was exploratory and should be interpreted cautiously given the limited number of failure events and wide confidence intervals.

Treatment decisions for ECR should not rely solely on the extent of resorption; factors such as symptoms, accessibility, and the presence of bone‐like tissue must also be considered, i. e. the practicality of treatment must be considered. Clinicians should recognize the limitations of radiographic evaluation, particularly in monitoring subtle changes that may reflect either progression or normal bone remodelling.

Further prospective clinical research is required to better understand the long‐term outcomes and sequelae of ECR treatment.

## Author Contributions

**Shanon Patel:** conceptualization, methodology, visualization, resources, original draft, writing, review and editing resources. **Nassr Al‐Nuaimi, Ryan Walsh, Paula Villa, Brent Hiebert, Elizabeth Perry** and **Rahul Sadhwani:** resources, editing, review.

## Conflicts of Interest

The authors declare no conflicts of interest.

## Data Availability

The data that support the findings of this study are available from the corresponding author upon reasonable request, subject to ethical and privacy restrictions.
